# Multi-modal comparison of murine and human incisal dentin-enamel junctions

**DOI:** 10.3389/abp.2025.14642

**Published:** 2025-08-18

**Authors:** Michael Truhlar, Sobhan Katebifar, Bradley Rosenberg, Roland Kroger, Alix C. Deymier

**Affiliations:** ^1^ School of Dental Medicine, UConn Health Center, Farmington, CT, United States; ^2^ Department of Biomedical Engineering, UConn Health Center, Farmington, CT, United States; ^3^ Department of Physics, University of York, York, United Kingdom

**Keywords:** dental Morphology, dentin, enamel, mechanical properties, micro-computed tomography

## Abstract

Human and mouse incisors are both primarily composed of dentin and enamel, which meet at an interface called the dentin-enamel junction (DEJ). However, incisors in the two species have very different growth patterns, structures, and loading requirements. Since the DEJ is responsible for minimizing cracking at this at-risk interface between mechanically dissimilar dentin and enamel, its structure is expected to be significantly different between humans and mice. Here, structural and compositional gradients across human and murine incisors DEJs were measured via microcomputed tomography and Raman spectroscopy. Density gradients across the DEJ were significantly larger in humans compared to murine teeth, likely due to the larger size of the mantle dentin. Multiple gradients in mineral content and crystallinity were found at the murine DEJ, while the human DEJ only exhibited gradients in mineral content. Models predicting the modulus across the DEJ according to compositional results show that mineral crystallinity is critical in regulating gradients in tissue stiffness across the murine DEJ. Together, these results show the multiple ways in which the DEJ can adapt to variations in the loading environment.

## Introduction

Most mammalian incisors consist of enamel and dentin as primary load-bearing tissues, with cementum covering the root to support periodontal attachment. Dentin is a porous tissue composed of approximately 70% mineral and 30% hydrated proteins most of which are collagen. The mineralized collagen fibers are organized radially around tubules which span from the pulp toward the enamel (Deymier-Black A. et al., 2014). The dentin contains a region with a significantly reduced number of tubules near the interface with the enamel which is described as mantle dentin (Ten Cate, 1980). Conversely, enamel is dense and acellular containing ∼96% mineral and 4% hydrated proteins, where the mineral hydroxyapatite crystallites are organized into rod-like structures (Ten Cate, 1980). These structural distinctions give rise to significant mechanical dissimilarities between the two tissues, with enamel being harder and stiffer but more brittle, while dentin is more compliant and fracture-resistant (Vaddi et al., 2023; Marshall et al., 1997; Ten Cate, 1980).

The interface between these two tissues, the dentin–enamel junction (DEJ), would be expected to be a site of stress concentration and failure due to the stark contrast in mechanical properties (Easson et al., 2024). However, in healthy dentition, the DEJ is exceptionally fracture-resistant, inhibiting crack propagation and tissue failure (Zhou et al., 2019). This exceptional interfacial toughness seems to arise because the DEJ is not a simple boundary, but rather a transitional zone with gradual changes in composition, microstructure, and mechanical properties (Easson et al., 2024; Zhou et al., 2019; Zaslansky, Friesem, and Weiner, 2006; Hasegawa et al., 2023; Fong et al., 1999a; White et al., 2000; Desoutter et al., 2023; Wang, Zhao, and Wang, 2021; Wingender et al., 2021). This gradient architecture is thought to enable effective bonding between the hard, brittle enamel and the softer, more resilient dentin, while minimizing stress concentrations during mastication. Understanding the nature of the DEJ is therefore essential for exploring its mechanical behavior and crack resistance (White et al., 2005). However, the complexity of this interface makes it difficult to obtain accurate measures of these DEJ gradients.

Establishing the width of the DEJ has been a challenge, resulting in variable methodology-dependent results due to its inherent structural complexity. In humans, its scalloped three-dimensional morphology complicates width measurements, especially when using conventional two-dimensional imaging techniques. The resolution limits of common microscopy methods and artifacts introduced during sample preparation can obscure the true extent of this interface. Macroscale mechanical testing on bovine teeth suggests an effective DEJ width of ∼100 µm (Zhou et al., 2019; Zaslansky, Friesem, and Weiner, 2006). Nanoindentation techniques have consistently found a DEJ size of 10–20 µm in human teeth (Hasegawa et al., 2023; Fong et al., 1999a) while micro-indentation reports values closer to 100 µm (White et al., 2000). Scanning Electron Microscopy (SEM) images show scalloped, micro-scalloped, and overlapping structures of the human DEJ, indicating a variation of composition across the DEJ at a scale of 1–3 µm (Desoutter et al., 2023; Wang, Zhao, and Wang, 2021; Wingender et al., 2021). Nano-scratch and x-ray spectroscopy identify gradients with values around 1–2 µm (Desoutter et al., 2023; Wang, Zhao, and Wang, 2021). This variability points to the need for a multi-modal investigation of DEJ structure and composition.

This complexity is further compounded by interspecies variation in the composition and structure of the DEJ (Möhring et al., 2023; Monson et al., 2020; Skinner et al., 2008; Kuczumow et al., 2021). This is especially important when animal models are used to study diseases of human dentition. Mice are widely utilized in dental research due to their modifiable genome, short gestation period, and well-characterized developmental pathways (Thesleff, 2003; Moore et al., 2024). However, murine dentition differs significantly from human dentition in both structure and growth patterns. Human incisors are non-growing, diphyodont teeth composed of a dentin core covered by an enamel crown, while mouse incisors are continuously growing (hypselodont) and asymmetric, with enamel present only on the labial surface and dentin on the lingual surface (Jiang et al., 2024; Tummers and Thesleff, 2003). These dissimilar incisors are used for a similar purpose of cutting food and other materials; however, murine teeth are made to wear down during feeding and gnawing behaviors. These differences are expected to lead to modified structure and mechanics of the DEJ. This study uses a multimodal approach to investigate changes in gradients across the DEJ of murine and human teeth and relate them to the tissue mechanics. The DEJ exhibits distinct compositional and mechanical gradients in human and murine incisors, and these gradients differ in width and structure due to species-specific variations in tooth morphology and function. We hypothesize that the DEJ in murine teeth displays a more abrupt transition zone compared to human teeth, reflecting adaptations to gnawing behavior, and that these differences contribute to altered mechanical properties and crack resistance across the interface. We hypothesize that the DEJ in murine teeth displays a more abrupt transition zone compared to human teeth and that these differences contribute to altering the mechanical properties and crack resistance across the interface, likely reflecting adaptations to promote whole tooth structural integrity while gnawing.

The development of mineral gradients across the DEJ is primarily driven by the biological processes of mineralization, which are tightly regulated by the activity of odontoblasts and ameloblasts. Odontoblasts in dentin secrete a collagen matrix that facilitates the initial deposition of minerals, while ameloblasts in enamel guide the highly ordered deposition of hydroxyapatite crystals (Bleicher et al., 2015; Imhof et al., 2020). The gradient formation results from varying mineral deposition rates and crystal size, with mineralization being regulated by matrix proteins like enamelin, amelogenin, and dentin matrix protein 1 (DMP1), which control both mineral content and crystal size (Moradian-Oldak and George, 2021). This phenomenon contributes to the interface’s graded mechanical properties. Understanding these gradients is essential to our study, which seeks to characterize how structural and compositional transitions at the dentin-enamel junction influence its mechanical function.

## Methods

### Sample preparation

Animal protocols were completed in accordance with the UConn Health Institutional Animal Care and Use Committee (protocol# AP-201383-1127). Lower incisors were dissected from five 4-5-month-old CD-1 mice (Charles River Labs, United States). Group size was determined using power analysis (β = 0.8, α = 0.05) and data from previous Raman studies on hard tissues (Moody et al., 2023; Easson et al., 2024; Vaddi et al., 2023). The teeth were then mechanically cleaned and stored at −4°C in Phosphate Buffered Saline (PBS) soaked gauze. Of the two incisors collected from each mouse, the lower left incisors were thawed and glued with Loctite gel control super glue onto a glass microscope slide with the DEJ perpendicular to the surface. Samples were polished utilizing silicon carbide sandpaper with grits from 2,000 to 10,000, followed by a Komet USA 94013F HP170 diamond embedded polishing wheel in a rotary tool set to 6,000 rpm. The remaining lower right incisors were wrapped in PBS-soaked gauze and frozen for microcomputed tomography (µCT).

One human left central incisor and one right lateral incisor, both mandibular, were provided from the archival collection of the Education Support Services Anatomy Laboratory at the University of Connecticut School of Dental Medicine on 15 June 2023, for the purpose of this research. These specimens had been in the possession of the institution for several decades and were fully anonymized. Exclusion criteria included the presence of caries or significant damage. There were no exclusions for either group or in any analysis. The two incisors were embedded in EpoKwick™ FC epoxy resin and mechanically polished using diamond slurry, progressing through decreasing grit sizes down to a final grain size of 0.1 µm to prepare flat, smooth surfaces for Raman spectroscopy. After collecting compositional data, the teeth were mechanically extracted from the epoxy and scanned via micro-CT to obtain structural information.

### Microcomputed tomography

**FIGURE 1:**
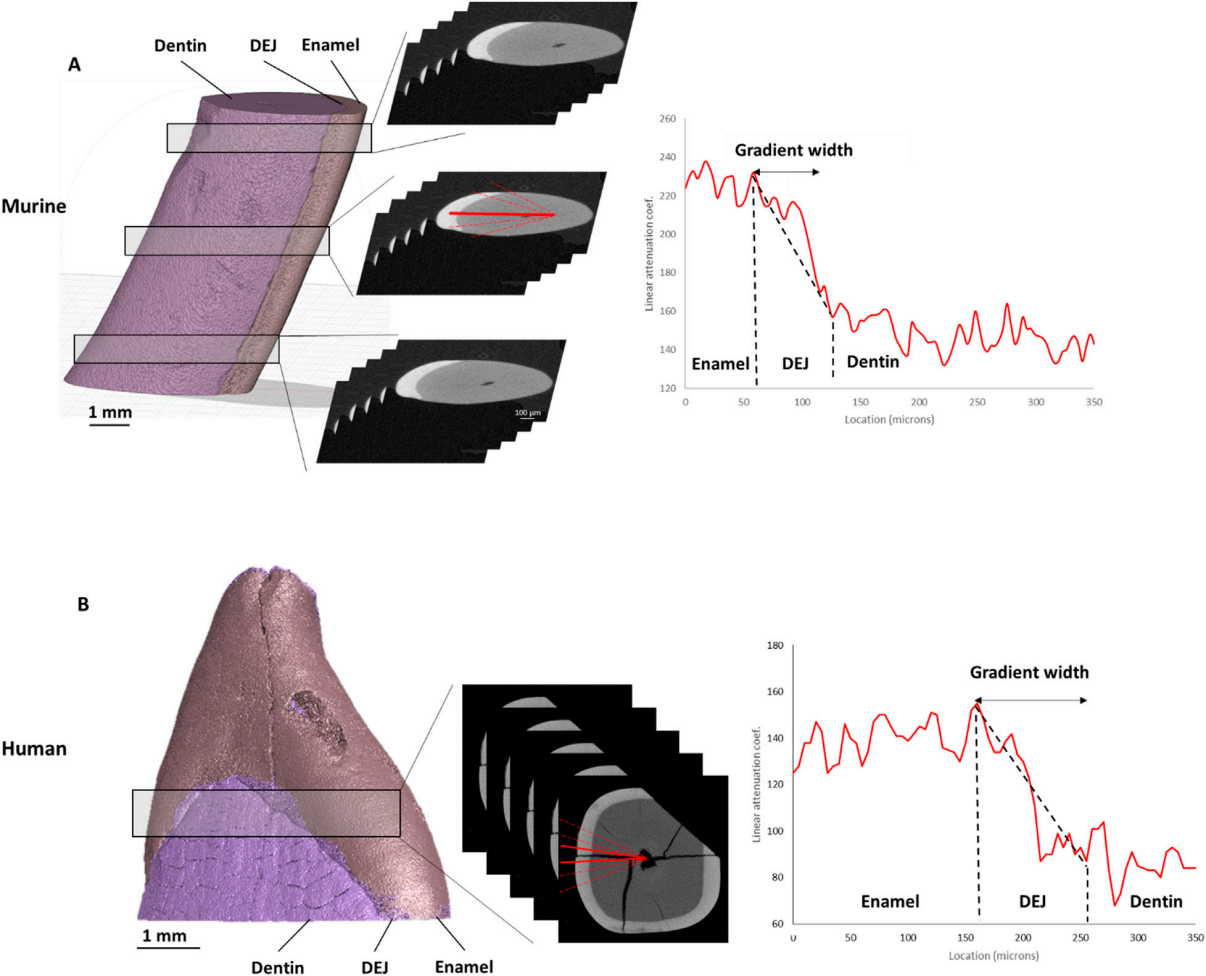
Density gradients from μCT. ROI locations are outlined—three for murine **(A)** and one for human **(B)** teeth. Corresponding representative plots of absorption intensity are shown, along with red lines drawn from the pulp cavity to the enamel surface. The average width of the DEJ density gradient for each tooth is presented on the right.

The lower right murine incisors (n = 5) and human incisors (n = 2) were imaged using a Scanco 50 micro-computed tomography (micro-CT) system. For the murine incisors, whole teeth were placed in the micro-CT system, and the midsection of each incisor spanning 7.3 mm was imaged at a resolution of 3.4 μm voxels with a source voltage of 55 kVp and current of 145 μA, (Figure 1). The crowns of both human incisors were scanned at 90 kVp with a current of 88 μA, to avoid super saturation in the enamel, with a resolution of 5 µm voxels. The difference in selected resolutions arises from the significant size difference between the murine and human samples. The murine samples were able to fit in the high-resolution Scanco sample tube, allowing for a 3.4 µm resolution, while the human teeth only fit into a Scanco sample tube, allowing for a 5 µm voxel size.

The cross-sectional images of the murine and human teeth were examined using the CTAn program (Bruker). For the murine samples, 5 sections were selected at 3 locations along the tooth: near the root apex (bottom), near the middle of the tooth (middle), and towards the incisal edge of the tooth (top). For each of these sections, 5 lines were drawn perpendicular to the DEJ from the pulp cavity to the enamel boundary (Figure 1). For the human teeth, 6 lines were collected across the DEJ in the mid-crown. The linear attenuation coefficient as a function of position along each line was then collected. These attenuation coefficient versus position plots showed increased attenuation in the enamel compared to the dentin, with a graded region at the DEJ. Since X-ray attenuation is related to the tissue density, we will refer to this gradient as a density gradient across the DEJ. These plots were fit in MATLAB to calculate the width of the gradient in absorption intensity data across the DEJ by fitting a logistic function to the intensity data and determining the full width half max (FWHM) of the derivative of that function.

### Raman spectroscopy scans

**FIGURE 2:**
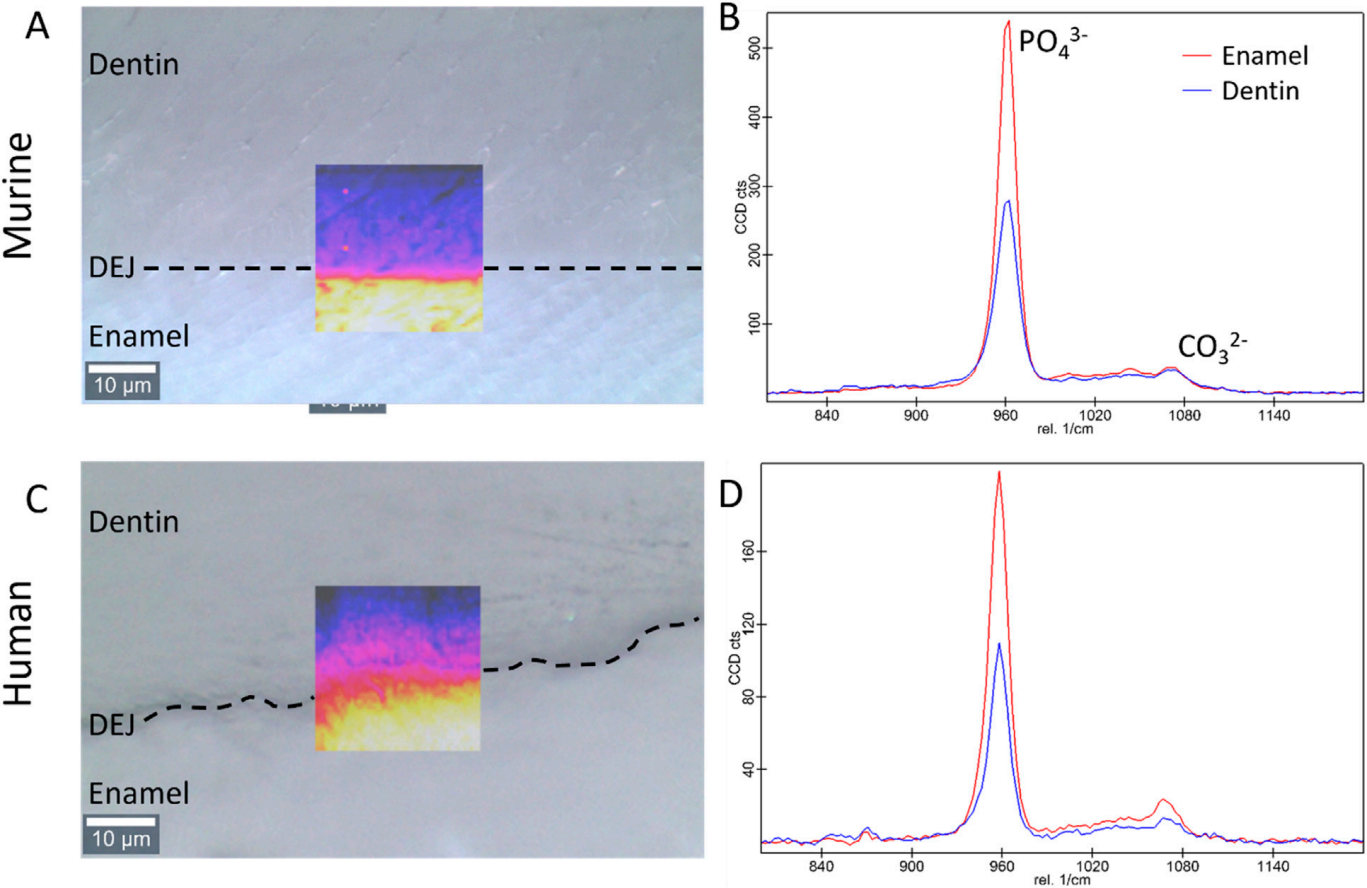
Bright field images of the dentin, enamel, and DEJ with an overlay of the Raman PO_4_^3−^ peak intensity in the region of interest for the **(A)** murine and **(C)** human teeth (transverse view). Representative Raman spectra showing the 960 cm^−1^ PO_4_^3−^ and the 1,070 cm^−1^ CO_3_^2−^ peaks in enamel and dentin for the **(B)** murine, and **(D)** human teeth.

Raman analysis was performed using a Witec Alpha 300 microscope paired with a Witec UHTS 400 spectrometer and a 785 nm laser. After identifying the location of the DEJ (Figure 2), we collected Raman area scans of the DEJ. For the murine samples (n = 5), one 25 × 25 μm^2^ area scan with a resolution of 0.5 µm was collected with an integration time of 20 s at the mid-point of the incisor length, similar to the middle region investigated in µCT. For the human incisors (n = 2), two area scans, one 25 × 25 μm^2^ with a 0.5 µm resolution and one 25 × 100 μm^2^ with a 1 µm resolution, were collected with a 20 s and 1 s integration time, respectively, at the midheight of the crown on the labial side near the region of interest selected in µCT. Although the resolution differs from that of micro-CT, region-of-interest (ROI) selection was carefully aligned across modalities to ensure consistent mapping and minimize discrepancies in density assessment. For human samples, Raman spectroscopy and micro-CT were performed on the same teeth, allowing for direct co-registration of ROIs. In contrast, for murine samples, Raman and micro-CT analyses were conducted on different teeth from matched experimental groups; therefore, mineral distribution patterns in mice represent an estimation across similarly prepared specimens rather than within the same tooth.

**FIGURE 3:**
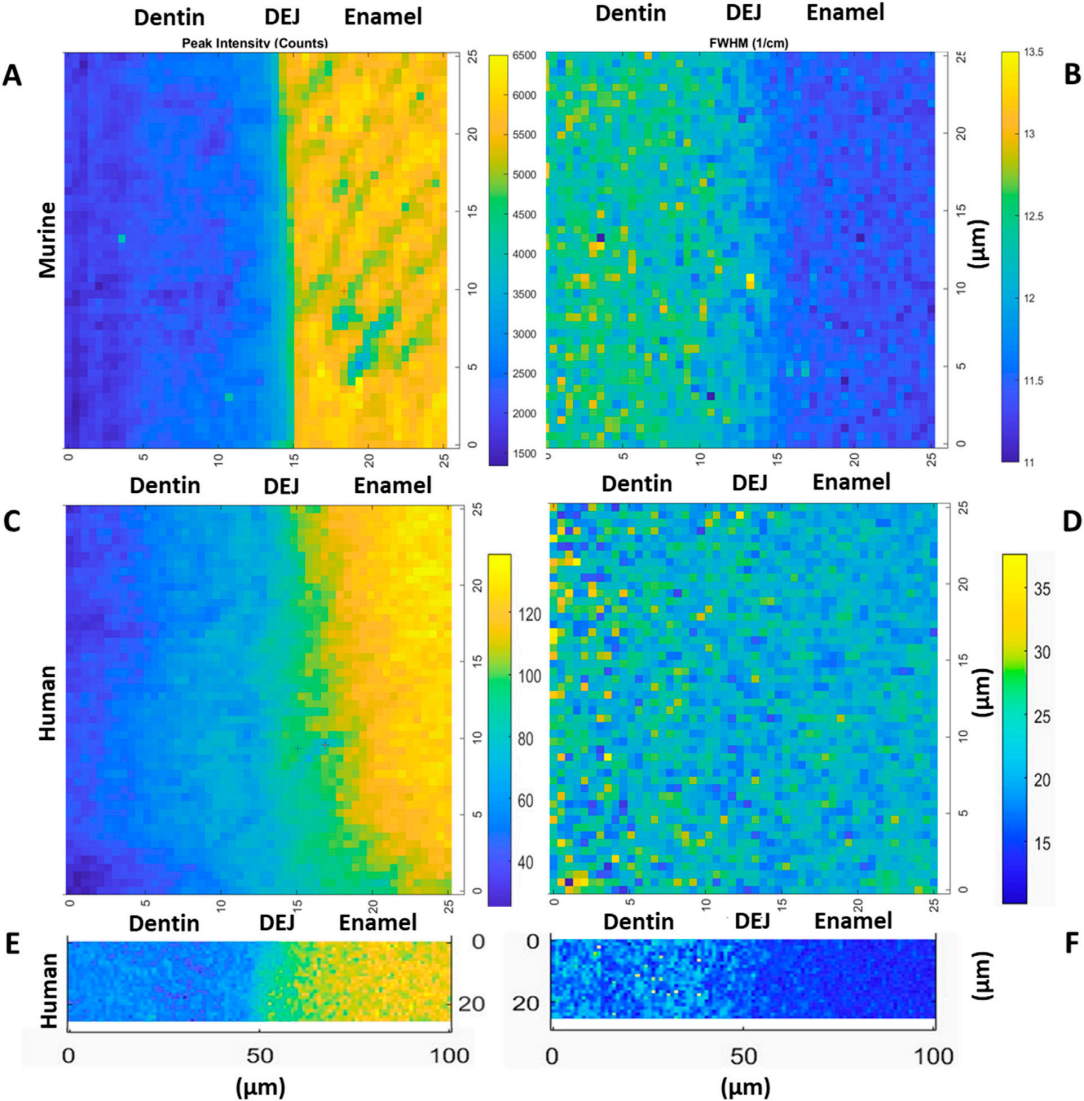
Representative maps of the 960 cm^−1^ peak intensity for murine incisors **(A)**, human incisors small area **(C)** and large area **(E)**, as well as the 960 peak FWHM for murine incisors **(B)**, human incisors small area **(D)** and large area **(F)**, (transverse view).

**FIGURE 4:**
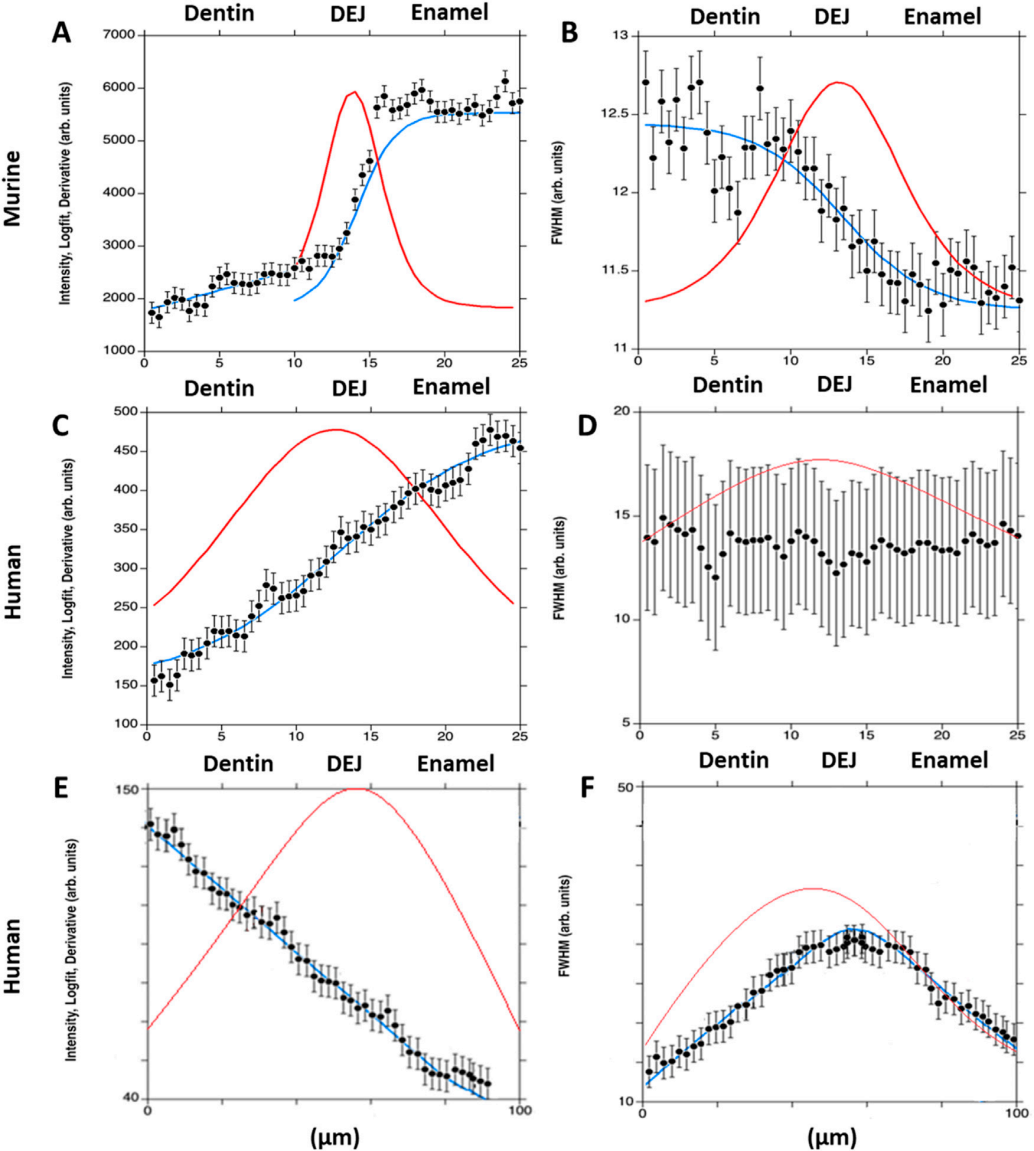
Plots show the 960 cm^−1^ peak intensity for murine incisors **(A)**, and human incisors—small area **(C)** and large area **(E)**; and the 960 cm^−1^ peak full width at half maximum (FWHM) for murine incisors **(B)**, and human incisors—small area **(D)** and large area **(F)**. A single-line fit was applied across the dentin–enamel junction (DEJ) in both human and murine samples. Solid blue lines represent the logistic fit, while solid red lines show its derivative, used to identify the gradient center and FWHM.

All the data from Raman spectra were corrected for cosmic rays and background subtraction using Witec Program 5.1. The 960 Δcm^−1^ phosphate peak and the 1,070 Δcm^−1^ carbonate peak were fit as a Lorentzian to obtain the peak max intensity, width, and position. Custom MATLAB code was then utilized to analyze the phosphate peak data. In summary, an experienced user was asked to provide a line perpendicular to the DEJ based on a 960 Δcm^-1^ phosphate peak intensity map. The orientation of this line was used to adjust the matrix, creating plots of peak intensity, FWHM, and peak position as a function of location. The max intensity, peak width, and peak position as a function of location across the DEJ were then fit for all lines utilizing a logistic regression. The derivative of this logistic fit was taken for each of our measurements to determine the center location of gradients (location of the max derivative value) as well as the width of the gradients of interest (FWHM of the derivative function), (Figures 3, 4). Values were averaged across all lines for each sample.

Based on the spatial profiles of phosphate peak intensity and carbonate content, mantle dentin was operationally defined as the ∼10–20 µm zone on the dentin side of the DEJ that exhibited a shallow, linear gradient in mineral content and mineral crystallinity. While traditional histological staining was not employed, this region was identified consistently across all murine and human samples based on its spectroscopic signature and location, consistent with known features of mantle dentin (Deymier-Black A. C. et al., 2014).

**FIGURE 5:**
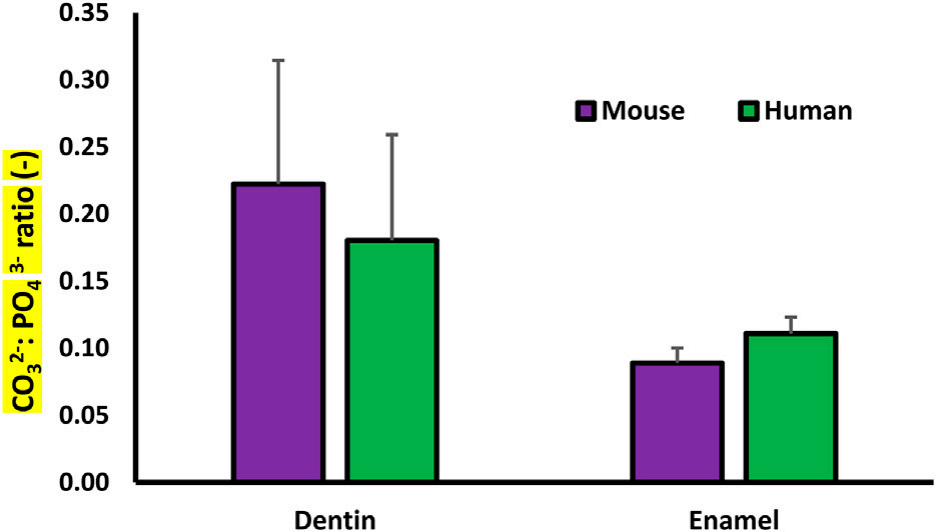
Plot of the Raman determined average Carbonate: Phosphate ratio for the dentin and enamel in the murine and human samples.

The ratio of the 1,070 to the 960 Δcm^−1^ peak max intensities, which represents the relative carbonate content in the mineral, was calculated at each point. These were then averaged across all lines parallel to the DEJ to obtain average CO_3_^2−^ content as a function of position across the DEJ. The first 20 points in the enamel and in the dentin were averaged to obtain the average mineral carbonate content in each of the tissues (Figure 5).

### Modeling DEJ mechanics via mineral gradients

To understand the contribution of changes in mineral content and crystallinity on the DEJ mechanics, we developed a simplified composite model of dental tissues. It was assumed that mineral content was proportional to the 960 peak maximum intensity and could be estimated from the average logistic fit of the 960 max intensity data. For the murine samples, both mineral content gradients were considered, and measures were made across 20 µm. For the human samples, the mineral gradient data from the 25 and 100 µm length scans were summed and considered over a span of 100 µm. Bounds were placed on this data such that pure dentin had a mineral content of 45 vol% (Fu and Kim, 2024; Ten Cate, 1980) and pure enamel, a mineral content of 90 vol% (Huaiquin-Zúñiga et al., 2024; Möhring et al., 2023; Hasegawa et al., 2023; Ten Cate, 1980).

The modulus of the tissue was calculated as a function of location, assuming that the material is loaded in isostrain. Although highly simplistic, especially for dentin, which is known not to exhibit one-dimensional alignment (Deymier-Black A. C. et al., 2014). This shows the maximum effect of changes in mineral content and modulus to provide an upper bound. A single representative sample was used for each sample type. We define crystallinity as being inversely proportional to the FWHM of the *ν*_*1*_-PO_4_ peak. Using previous experimental data relating crystallinity to modulus (Wingender et al., 2021; Deymier et al., 2017), a linear correlation was established between the width of the *ν*_*1*_-PO_4_ peak and the mineral modulus. The logistic fit of the *ν*_*1*_-PO_4_ FWHM data was used as a measure of the change in FWHM as a function of position across the DEJ. These data were used to calculate the mineral modulus as a function of position.

Both sample types had gradients in *ν*_*1*_-PO_4_ FWHM and maximum intensity; these values were integrated into the isostrain calculations described above to determine the modulus of the overall tissue as a function of position, while accounting for contributions from the mineral crystallinity and content. From this, we predicted the change in modulus based on crystallinity as a function of position across the DEJ. Using a span of 20 and 100 μm for the murine and human teeth, respectively, the total tissue modulus was estimated by averaging the varying moduli across the tissue. Even if mineral content is estimated from Raman intensity, mechanical modulus analysis reveals how that content, in combination with crystallinity, governs tissue function, especially in a mechanically graded structure like the DEJ.

### Statistical analysis

Gradient widths are reported as mean values ± the standard deviations. Comparisons between gradient widths were performed using ANOVA, followed by means comparison testing using Tukey tests. Significance was set at p < 0.05.

## Results

### Density gradients from μCT

**FIGURE 6:**
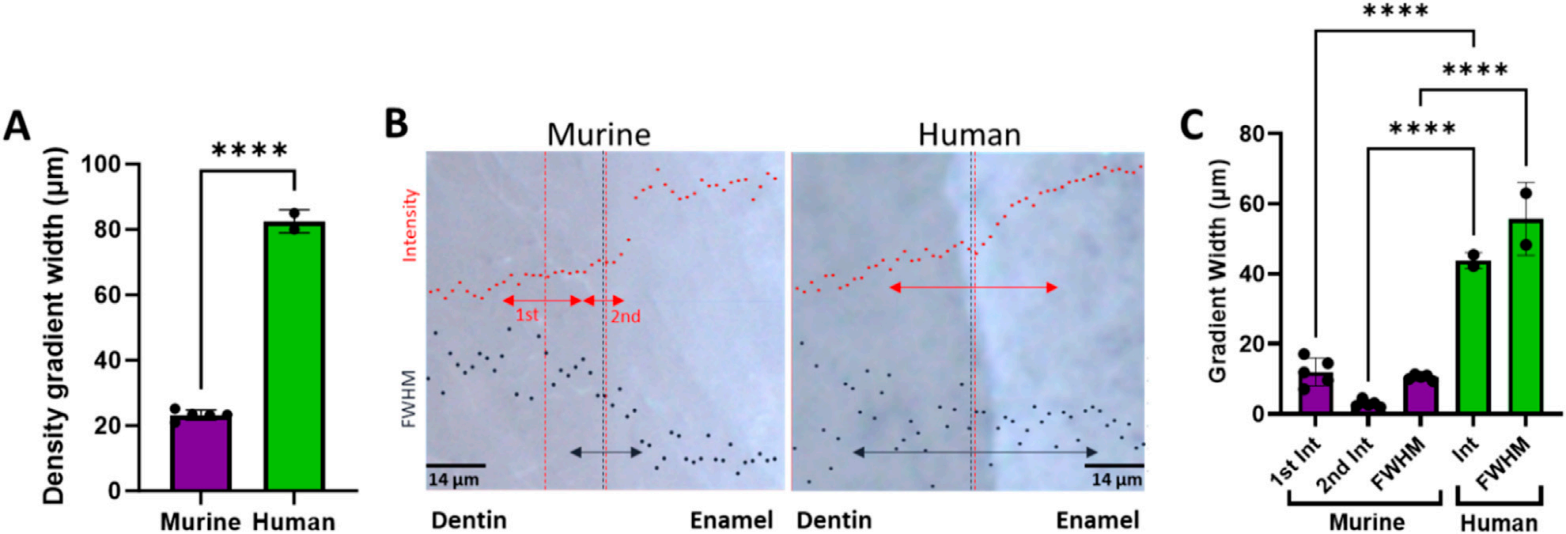
**(A)** Plot comparing the average murine density gradient width as determined from µCT to the human average. **(B)** Overlays of representative 960 peak intensity (red) and 960 peak FWHM (black) in bright-field images of murine and human DEJs (transverse view). Arrows represent the width of the measured gradients, while the vertical dotted lines represent the center of the respective gradients. **(C)** Plot of the Raman spectroscopy measured gradient widths. ****represents p ≤ 0.0001.

For the murine teeth, gradients in the tissue density as determined from micro-CT attenuation coefficients were measured across the DEJ. The average width of the density gradients across the DEJ was 24.4 ± 0.72 μm at the bottom (towards the root apex), 22.89 ± 2.44 μm at the middle, and 22.33 ± 3.34 μm at the top (near the incisal edge) of the tooth (Figure 1). Density gradient width was not significantly different as a function of location. The average density gradient width was 23.12 ± 2.48 μm in murine teeth, whereas the mid-crown region of human incisors exhibited a significantly broader gradient of 82.5 ± 3.5 μm (Figure 6A).

### Phosphate peak and FWHM gradients

The gradient widths for phosphate peak intensity and phosphate peak full width at half max (FWHM) are correlated with phosphate content (peak intensity) and mineral crystallinity (inverse of the FWHM), respectively (Figure 3). Visual inspection of the murine area scans indicated the presence of two distinct 960 cm^−1^ peak intensity gradients across the DEJ: an initial shallow gradient (1st Max Int), followed by a second steeper gradient (2nd Max Int), (Figures 6B,C). It thus became necessary to fit each gradient independently.

In the murine incisor, the first maximum intensity (Max Int) gradient was shallow and best described by a linear regression, with an average width of 12.0 ± 3.9 μm. In contrast, the steeper second Max Int gradient followed a logistic fit, as previously described, and exhibited a narrower width of 2.9 ± 1.0 μm. The FWHM gradient had an intermediate width of 10.5 ± 0.8 μm. A statistically significant difference was observed among the widths of the three gradients (p < 0.05). Spatially, the first Max Int gradient was shifted 7.6 ± 1.2 μm toward the dentin relative to the second Max Int gradient. However, the second Max Int and FWHM gradients were co-localized, with an average positional difference of only 0.11 ± 0.45 μm.

The human incisors exhibited a very different gradient geometry (Figures 4, 6B,C). Both the small and large area scans, 25 and 100 μm, respectively, exhibited a single gradient in mineral content. Due to differences in scanning resolution, the measured mineral content gradients varied, with narrower widths observed in small area (25 µm) scans (22.9 ± 1.7 µm) and broader gradients in large area (100 µm) scans (43.8 ± 2.3 µm). The peak FWHM did not exhibit a gradient in crystallinity in the 25 µm scan, suggesting that this gradient may be occurring over a long length scale. This was supported by the fact that a gradient in FWHM well fit by a logistic function with a width of 63.5 ± 1.3 µm was clearly seen across the DEJ in the 100 µm scans. Once again, the center of the FWHM gradient is co-localized with the gradient in mineral content.

### Dentin and enamel carbonate content

The average CO_3_^2−^:PO_4_^3−^ ratio for minerals in the enamel and dentin was collected from each of the Raman data sets. The average CO_3_^2−^:PO_4_^3−^ ratio for the dentin mineral was significantly higher than that of the enamel for both the murine and human samples. There was no significant difference between the carbonate content of the murine and human samples (Figure 5).

### Theoretical predicting of the elastic modulus

**FIGURE 7:**
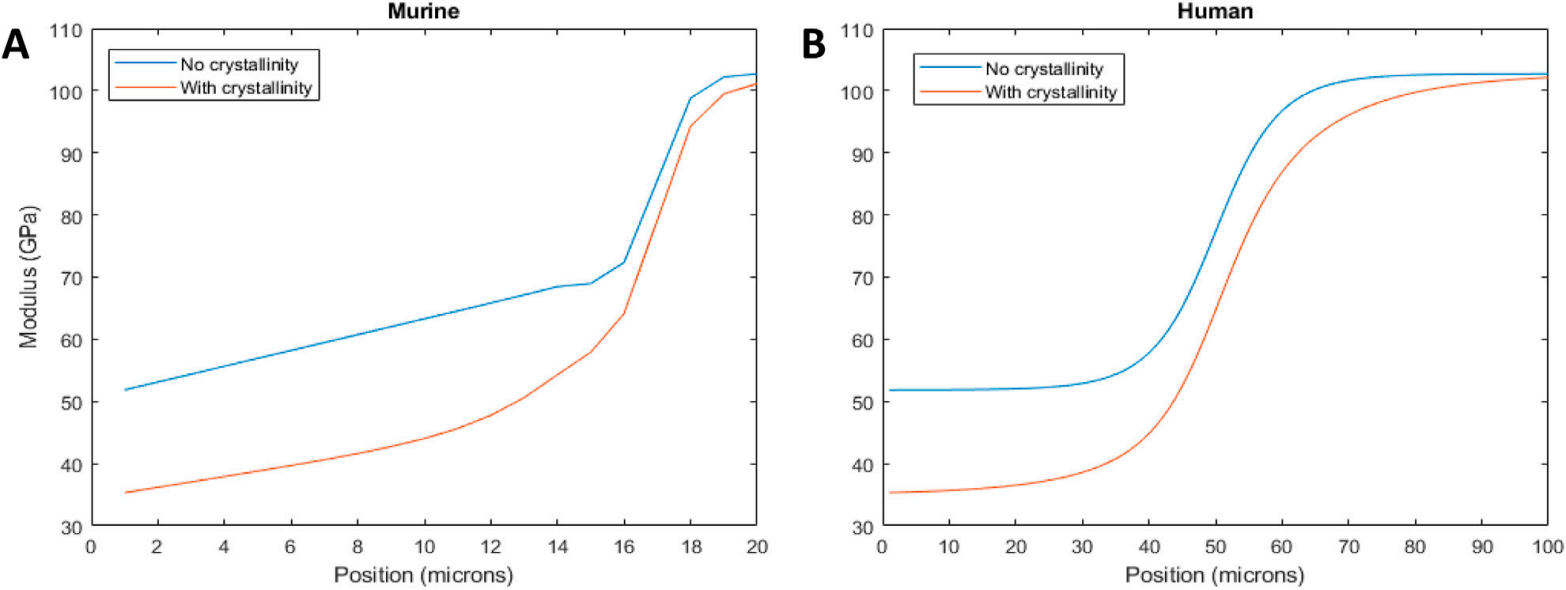
Tissue modulus as a function of position across the DEJ, considering the mineral content gradient (blue line) and crystallinity (red line) for murine **(A)** and human **(B)** incisors.

For the murine samples, plots of the tissue modulus as a function of position based on the 960-peak intensity as an indicator of mineral content showed that the tissue modulus changed with position across the gradient (Figure 7). The starting dentin modulus was 51.9 GPa, higher than generally accepted values for dentin (Zhang et al., 2014), most likely because porosity is not accounted for in the isostrain model. The enamel had a modulus value of 102.7 GPa, in agreement with many mechanical measures (Zhang et al., 2014). When considering crystallinity, the dentin modulus decreased to 35.3 GPa, much closer to the commonly measured values of 20–25 GPa, where the difference is likely due to the presence of porosity and non-isostrain loading of the dentin. The enamel value remained elevated at 100.1 GPa. The modulus gradient width increased when considering crystallinity. The average modulus was reduced by 15% from 72.1 GPa to 62.7 GPa when considering both crystallinity and mineral content.

For the human incisor, the starting and ending moduli are the same as the murine samples, since identical assumptions have been made about the mineral content. However, consideration of the FWHM resulted in a widening of the mechanical gradient. The wider mechanical gradient when crystallinity was considered reduced the overall tissue modulus, across the 100 microns from 76.8 GPa to 66.9 GPa, a decrease of 13%.

## Discussion

Despite having the same building blocks, mouse and human incisors exhibit different structural organization, growth patterns, and loading regimes. One would therefore expect the DEJ to exhibit modified structures adapted to each case. In this study, we present the first direct comparison of the DEJ structures between murine and human incisors, highlighting key differences in gradient width, composition, and organization. By analyzing these structural variations, we explore their potential implications for mechanical performance, including stress distribution and resistance to fracture at the DEJ interface.

The dentin-enamel junction (DEJ) width has been reported between ∼2 and 100 μm, depending on location, species, and mode of measurement (Zhou et al., 2019; Zaslansky, Friesem, and Weiner, 2006; Hasegawa et al., 2023; Fong et al., 1999a; White et al., 2000; Desoutter et al., 2023; Wang, Zhao, and Wang, 2021; Wingender et al., 2021). Here, µCT and Raman spectroscopy were used to identify structural and compositional gradients specifically across the dentin–enamel junction (DEJ) in both human and mouse incisors. The density gradient, which correlates with tissue mineralization and overall density (Souleyman et al., 2021), was localized to the DEJ region and found to be approximately four times wider in human incisors (∼80 µm) compared to murine incisors (∼20 µm). This finding highlights species-specific differences in the spatial extent of the DEJ and its associated mineral transitions. This wider gradient in humans may be indicative of hyperallometric scaling since human incisors are 7-9X wider than murine incisors (Shuhaibar et al., 2021; Alqahtani et al., 2021; Jiang et al., 2024). However, the larger width may also be related to changes in the mantle dentin between humans and mice. Mantle dentin, a tissue found between the DEJ and the circumpulpal dentin (Ten Cate, 1980), has often been considered a part of the DEJ tissue (Desoutter et al., 2023; Zaslansky, Friesem, and Weiner, 2006). Our Raman results do suggest that there are differences in the width of the non-mantle dentin DEJ, as shown by the narrow gradient in mineral content. However, these differences do not explain the fourfold increase in density gradient width in humans compared to mice (Figure 4). Therefore, we propose that the wider density gradient in humans is indicative of a wider mantle dentin region.

Studies of the DEJ using Raman spectroscopy have reported a wide range of gradient widths in human teeth, typically between ∼4 and 50 µm (Xu et al., 2009; Alebrahim et al., 2015; Desoutter et al., 2023; Gallagher et al., 2010). In our study, we observed a single mineral content gradient width on the order of 20–40 µm in human incisors, depending on measurement resolution. This value is larger than those reported by some previous studies (Gallagher et al., 2010; Xu et al., 2009; Desoutter et al., 2023; Alebrahim et al., 2015), but closely aligns with findings from Slimani et al., 2017, particularly in third molars. Furthermore, our results are consistent with nano-indentation studies on human incisors, which reported gradient widths ranging from 15 to 25 µm (Wingender et al., 2021). The variation in reported gradient widths across studies can likely be attributed to differences in tooth type (molars vs incisors), sample preparation methods, spatial resolution of the techniques employed, and criteria used to define the gradient boundaries. For example, studies using lower spatial resolution or broader compositional definitions may report narrower gradients, whereas higher-resolution techniques like ours, focused specifically on mineral content variation, can capture more detailed transitions. This is clearly seen by the difference in gradient width measured in the same area via Raman using two different resolutions. Additionally, anatomical variability and age-related changes in mineralization may further contribute to discrepancies in measured DEJ widths. Overall, our findings fall within the upper range of previously reported values and support the presence of a distinct mineral gradient at the DEJ in human incisors.

Both the murine and human samples exhibited gradients in mineral crystallinity across the DEJ. These crystallinity gradients co-localized with the gradient in mineral content (in the case of the murine samples, the 2nd steeper mineral content gradient). This co-localization suggests that both processes are regulated by cellular deposition of dentin and enamel at the DEJ during development. Interestingly, in the human samples, this crystallinity gradient was significantly wider than the gradient in mineral content, with an average value greater than 60 µm. This is in agreement with Slimani et al., 2017, who reported that the crystallinity gradient in human teeth extended over a broader region than the gradients in mineral or collagen content, spanning between 40 and 80 µm. The murine samples similarly exhibited a crystallinity gradient that was wider than the steeper 2nd gradient in mineral content, with an average value around 10 µm. This discrepancy between the gradient in amount of mineral versus mineral maturity suggests that despite their similar origination at the DEJ location, their maturation and development undergo different pathways. Tissue mineral content is primarily cell-mediated with minimal changes over time; however, mineral crystallinity is ever-evolving thanks to physiochemical processes like Oswald ripening, a phenomenon where smaller, less stable crystals dissolve and redeposit onto larger, more stable ones over time (Boskey and Coleman, 2010). This difference may explain the strong difference in gradient width. Unlike the single mineral content gradient observed in humans, mice exhibit a two-part pattern: an initial shallow linear gradient spanning approximately 12 μm, followed by a rapid increase over the next ∼3 µm. This second gradient is on the order of measurements made using high-resolution imaging (Desoutter et al., 2023; Wang, Zhao, and Wang, 2021). The total width of the first and second gradients approaches the width of the density gradient measured in µCT. This suggests that the first gradient, present on the dentin side of the DEJ, is associated with the mantle dentin, while the 2nd gradient is a measure of the mineral content gradient across the DEJ interface.

Changes in mineral composition and crystallinity affect the mineral stiffness (Wingender et al., 2021; Deymier et al., 2017; Xu et al., 2012). For example, carbonate substitutions in apatite reduce crystallinity and, in turn, mineral stiffness by up to 50% (Wingender et al., 2021; Deymier et al., 2017). Apatite mineral in dentin contains approximately three times the amount of carbonate as enamel apatite (Kuczumow et al., 2021), possibly explaining the crystallinity gradient. We use that knowledge to determine the contributions of gradients in mineral content and mineral crystallinity on the mechanics of the DEJ. First, we calculated the modulus as a function of position, due to mineral content only, for human and murine incisors (Figure 7). Both show an increase in modulus with mineral content from the dentin to the enamel. The width of these gradients is proportional to the changes in *ν*_*1*_-PO_4_ peak height across the DEJ, and therefore, the width of the modulus gradient is larger in the human samples than in the murine samples [Note the difference in X-axis between the two plots (Figure 7)]. The average DEJ modulus (over 20 µm for mice and 100 µm for humans) is 72.1 GPa and 76.8 GPa, respectively, suggesting that despite the differences in structure, the overall modulus is preserved. However, when we consider murine and human crystallinity gradients, the average modulus drops to 62.7 GPa in murine and 66.9 GPa in human; a reduced interfacial modulus is associated with increased crack absorption, suggesting that the crystallinity gradient may prevent DEJ cracking (Broomell et al., 2006; Easson et al., 2024; Vaddi et al., 2023). Consideration of the mineral crystallinity also widens the mechanical gradient across the DEJ, which should increase resistance to fracture. This suggests that the DEJ has developed structures to prevent fracture along this interface. Here we see the importance of crystallinity gradients in controlling the mechanical properties of interfaces between mineralized tissues (Kegulian et al., 2024; Easson et al., 2024; Wang et al., 1998).

The observed differences in DEJ gradient widths between murine and human samples are influenced not only by relative tooth size but also by species-specific developmental and structural differences. The murine gradients are consistently smaller than those seen in the human samples. This may be primarily allometric and accounting for the increased loads felt by human dentition. However, the distinct difference in the distribution of mineral content between the two species suggests that there are also functional adaptations. Murine teeth exhibit continuous growth and a need for controlled fracture during gnawing. The presence of an abrupt mineralization gradient at the DEJ would allow for increased stress concentrations and fracture risk, allowing for enamel fracture (Zhou et al., 2019). Meanwhile, the 1^st^ linear gradient, which we theorize is associated with mantle dentin, could serve as a shock absorber during usage. Human dentition, on the other hand, is permanent and therefore benefits from wider gradients, allowing for rendition of the enamel and reduced cracking.

This study has some limitations. First, the sample size was relatively small especially in the case of the human samples, which may limit the generalizability of the findings. Secondly, due to the differences in techniques, it was not possible to make full correlative measures between the µCT and Raman measurements; however, we were careful to select similar regions in order to minimize variation. Finally, the mechanical model proposed here is simplistic, establishing a direct correlation between mineral composition, crystallinity, and stiffness and assuming isostrain loading. In reality, local microstructural factors—such as collagen orientation in dentin, crystal alignment in enamel, and the presence of residual organic material—may further modulate the tissue modulus. However, the result shown here suggests that even in the absence of complex anisotropic modeling or explicit organic matrix characterization, the gradients in mineral content and crystal structure are sufficient to establish a mechanical transition zone that may help dissipate stress and prevent failure at this interface. In the future, hierarchical mechanical studies integrating site-specific measurements, such as nanoindentation and atomic force microscopy (AFM) could provide a more comprehensive validation of the predicted modulus profiles and refine our understanding of structure–function relationships at the DEJ.

### Conclusion

Raman spectroscopy and µCT were used to measure compositional and structural gradients across DEJs from mouse and human incisors. Compared to mice, humans showed larger gradations in tissue density, mineral content, and mineral crystallinity across the DEJ. In addition, murine samples exhibited a two-part mineral content gradient with an abrupt interface that abuts a shallow linear gradient, which is theorized to be associated with the mantle dentin. Gradients in mineral content and crystallinity were used to predict the DEJ moduli as a function of position. It was shown that gradients in mineral crystallinity lead to a reduction in the overall tissue modulus and broadening of the mechanical gradient when compared to predicted values, only accounting for mineral content. These results highlight the importance of considering structural and compositional factors when predicting the mechanical behavior of interfacial mineralized tissues.

## Data Availability

The raw data supporting the conclusions of this article will be made available by the authors, without undue reservation.
